# Morphology of the male reproductive system and sperm ultrastructure of the green lacewing, *Chrysopa pallens* (Rambur, 1838) (Neuroptera: Chrysopidae)

**DOI:** 10.1186/s40850-023-00175-8

**Published:** 2023-08-28

**Authors:** Xiaoping Liu, Xingkai Guo, Yanjiao Feng, Lisheng Zhang, Mengqing Wang, Yuyan Li, Jianjun Mao

**Affiliations:** State Key Laboratory for Biology of Plant Diseases and Insect Pests, Key Laboratory of Natural Enemy Insects, Ministry of Agriculture and Rural Affairs, Institute of Plant Protection, Chinese Academy of Agricultural Sciences, No. 2 West Yuanmingyuan Rd., Haidian District, Beijing, 100193 P. R. China

**Keywords:** *Chrysopa pallens*, Testis, Spermatid, Spermatozoa, Accessory gland

## Abstract

**Background:**

*Chrysopa pallens* is one of the most beneficial and effective natural predators, and is famous for its extensive distribution, wide prey spectrum, and excellent reproductive performance. This study examined the anatomy and fine structure of the *C. pallens* reproductive system and spermatogenesis.

**Results:**

The male reproductive system of *C. pallens* comprises a pair of testes, a vas deferens, seminal vesicles, accessory glands, and short ejaculatory ducts. The testes were already mature on the day of emergence, but the accessory glands did not mature until 5 days post-emergence. In early spermatids, the flagellum had an axoneme on one side of the two mitochondrial derivatives. The nucleus was surrounded by parallel crystalline and paracrystalline materials. The spermatid envelope extends towards the paracrystalline material in a tail-shaped wing. In mature spermatids, the axoneme is located between the two accessory bodies and mitochondrial derivative sets. The parallel-crystalline and paracrystalline materials disappeared. In the testes, the wall of seminal cysts consists of a layer of epithelium, a muscular-connective sheath, and several vesicles of different sizes. The mature seminal cysts contained 128 spermatozoa. The accessory gland is composed of six parts: ventral papilla-like protuberance, anterior glandular lobe, lateral glandular lobe, seminal cyst, posterior kidney-shaped lobe, and posterior papilla-like protuberance. Muscle fibers and secretory granules are extensive.

**Conclusions:**

This study provides information on the reproductive system of *C. pallens* and offers a resource for taxonomy and reproductive biology.

## Introduction

Insects are considered extremely successful animals, at least partly because of their efficient reproductive capacity [1]. The reproductive system of insects comprises a collection of genitalia and internal reproductive organs that play several roles in producing progeny. The genitalia reside in the appendage of the abdominal genital segments during copulation, insemination, and oviposition, and are known as copulatory organs for males or ovipositors for females [2]. In contrast to the genitalia, the internal reproductive organs are derived from the ectoderm or mesoderm and consist of several parts with sexual differences. In insects, male internal reproductive organs usually include a pair of testes, a pair of vas deferens, a pair of accessory glands, a pair of seminal vesicles, and an ejaculatory duct [3, 4]. However, the reproductive systems of insects vary in appearance, position, and number among different groups. Furthermore, the morphology of organs, tissues, and organelles in the reproductive system may vary among different orders and even among different species [3, 5–11].

Morphological and ultrastructural features of the genital system also offer convincing evidence for studies in systematics, reproductive biology, and evolutionary biology [2]. Over the past several decades, studies on insect reproductive systems have mainly focused on Diptera, Hemiptera, Coleoptera, Hymenoptera, and rare insect orders such as Mecoptera and Zoraptera [2, 4, 6–14]. Neuropterans include several important predatory species with a promising application potential in the biocontrol of insect pests. However, the phylogenetic analysis of this order is challenging because of the large number of families in Raphidioptera and Megaloptera. To date, the relationships among Hemerobiiformia, a suborder within the Neuropteran taxon, remain debated [15, 16]. Comparative analysis of the sperm structure of different insect orders has demonstrated that sperms have a series of features that are useful for elucidating the relationship between insect taxa. In the spermatozoa of Neuroptera species such as *Eumantispa harmandi* and *Chrysopa formosa*, the nucleus is encircled by a nuclear envelope that, in its anterior domain, fans out laterally into one wing. *C. formosa* has two large mitochondrial derivatives that encircle a conventional axoneme in a 9 + 9 + 2 microtubular pattern. The anterior sperm region is surrounded by dense external material [16, 17]. Lacewings are well-known for their symbiotic relationships with microbes, including bacteria and filamentous fungi. In the green lacewing *Chrysoperla carnea*, all stages of microsporidium develop in direct contact with the host cell cytoplasm. Microsporidian spores were observed in cells of the proventriculus, diverticulum and in epithelial cells of the posterior midgut [18, 19].

The green lacewing, *Chrysopa pallens*, is one of the most beneficial and effective natural predators, famous for its extensive distribution, wide prey spectrum, and excellent reproductive performance [20, 21]. In the present study, we examined the development and morphology of the male *C. pallens* reproductive system using light transmission electron microscopy (TEM).

## Materials and methods

### Insects

*C. pallens* was fed pea aphids (*Acyrthosiphon pisum*) and maintained at 25 ℃ and 70% relative humidity (RH) under a 16:8 (L:D) h photoperiod. The male and female adults were kept together after emergence to ensure complete mating.

### Dissection and light microscopy

Male adults at different developmental stages were anesthetized with diethyl ether and dissected in 0.1 M phosphate-buffered saline (PBS, pH 7.4). Optical microscopy of the testes, vas deferens, and accessory glands was performed on fresh samples at 20-fold magnification (VHX-2000; KEYENCE, Germany).

### Transmission Electron Microscopy (TEM)

Five days post-emergence, males were dissected in 0.1 M PBS. The testes and accessory glands were fixed in 2.5% glutaraldehyde plus 3% sucrose in PBS buffer at 4 ℃ for 12 h. The samples were washed three times for 15 min each with PBS and fixed in 1% osmic acid for 2 h. The samples were dehydrated in a graded ethanol series (50%, 70%, 80%, 90%, and 95%) for 15 min each, then in 100% ethanol for 30 min, and finally 100% acetone for 30 min. The samples were infiltrated with a mixture of acetone and Spurr resin (1:1 v/v) for 1 h, followed by a 1:3 mixture for 3 h, and finally with Spurr resin for 5 h. The samples were embedded in fresh Spurr resin at 70 ℃ for 5 h. A Reichert-Jung Ultracut E ultramicrotome (Reichert, Vienna, Austria) was used to prepare ultrathin sections of 70–90 nm. After staining with 1% uranyl acetate for 15 min, ultrathin sections were examined under a Hitachi HT7700 transmission electron microscope (Hitachi, Tokyo, Japan).

## Results

### General morphology of male reproductive system

**Fig. 1:**
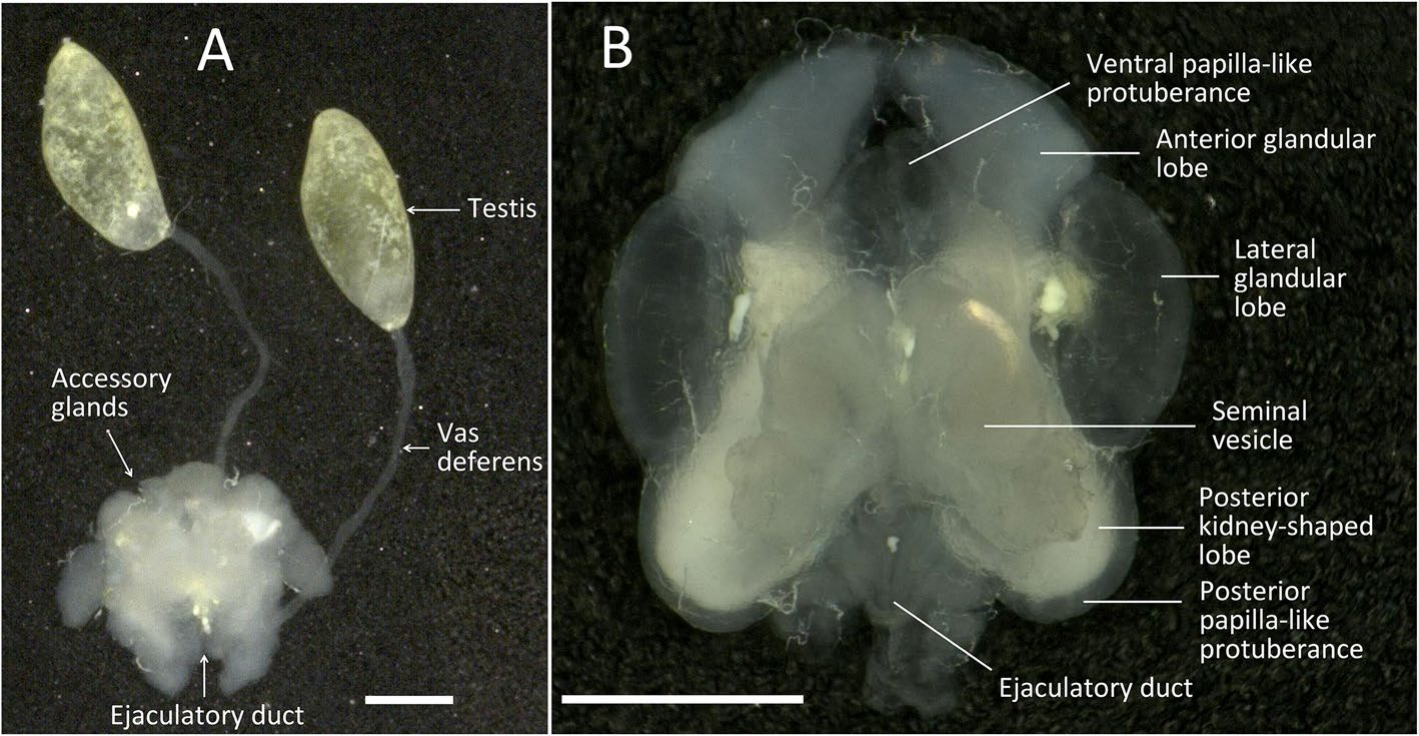
The male reproductive system of *C. pallens* and the structure of its accessory glands. **A** The male reproductive system of *C. pallens* (15-day-old). **B** Ventral view of the accessory glands (6-day-old). Bars = 500 μm

The male genital system of *C. pallens* consists of two symmetrical egg-shaped testes, a pair of vas deferens, seminal vesicles, accessory glands, and an ejaculatory duct (Fig. 1A). Each testis was kidney-shaped and approximately 1.2 mm in length and 440 μm in width. They were joined to the seminal vesicles by a long and slender vas deferens (approximately 2.5 mm) at the distal end. Seminal vesicles are located in the outermost ventral region of the abdomen. The accessory glands were milk-white in color, symmetrical and approximately 1.2 mm in diameter. Each half consists of six lobes on all sides, including the ventral papilla-like protuberance, anterior glandular lobe, lateral glandular lobe, seminal vesicle, posterior kidney-shaped lobe, and posterior papilla-like protuberance. The middle part of the accessory gland was thicker than the surrounding area. The ventral papilla-like protuberance, lateral glandular lobe, and posterior papilla-like protuberances are semi-transparent; however, the anterior gland lobe, seminal vesicle, and posterior kidney-shaped lobe are condensed. The seminal vesicle is ventral to and fused to the accessory gland. The ejaculatory duct was short and was embedded in the space between the two posterior ends of the accessory glands (Fig. 1B).

**Fig. 2:**
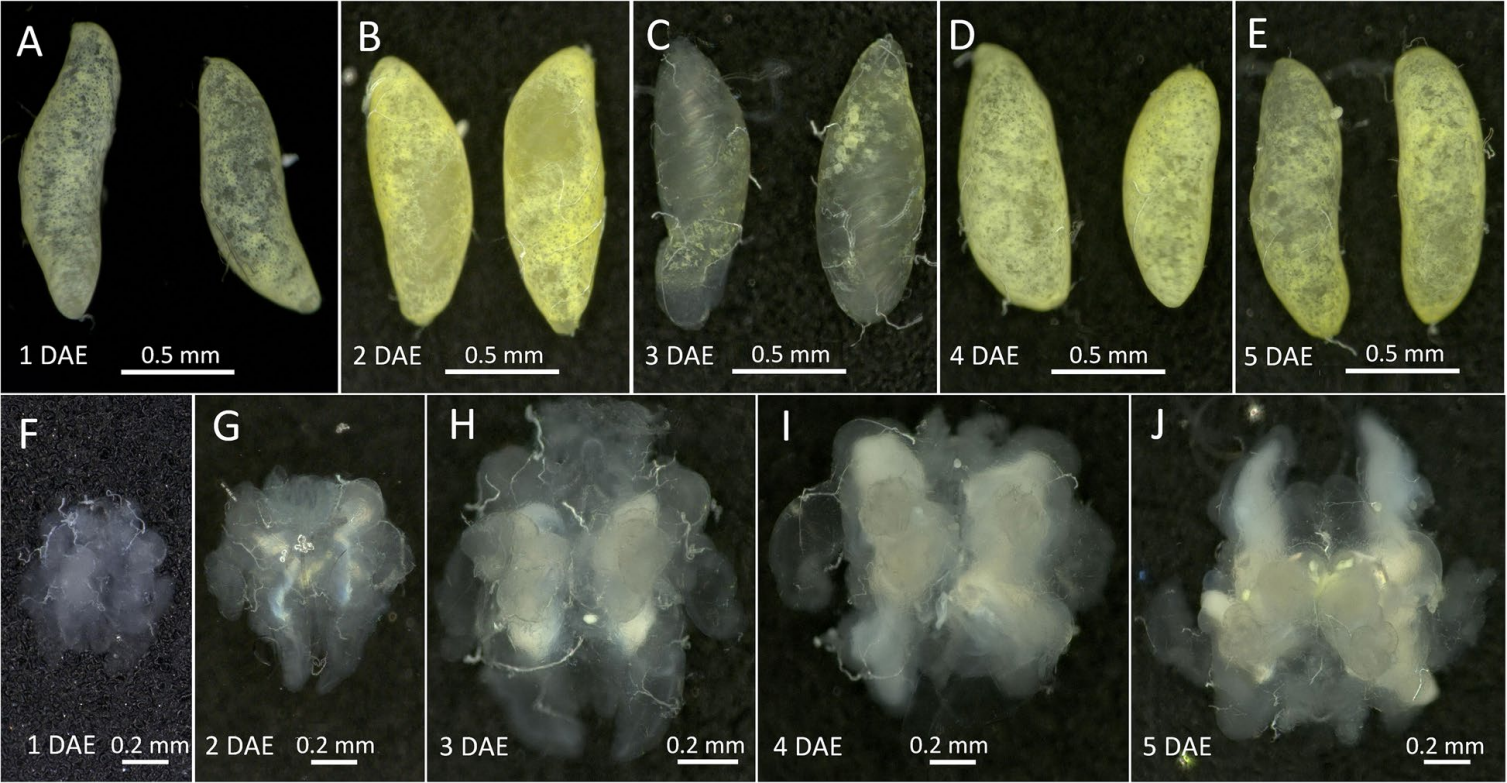
Developmental process of *C. pallens* male reproductive system. The *C. pallens* male reproductive system at different developmental stages was shown. The testes in adults 0 days post-emergence (DAE) have a similar size and shape to that in older adults (**A-E**). The accessory glands in 0-day-old male adults were small in size and then grew gradually with the increase of age and became fully mature 5 DAE (**F-J**).

The testes were fully developed 1 d post-emergence (DAE) and showed a similar size (approximately 1 mm long, 0.4 mm wide) and morphology to that of older male adults (Fig. 2A-E). However, the accessory glands exhibited marked differences in size and appearance between newly emerged and old males. The accessory glands at 1 DAE were in the initial developmental stage and smaller than those at 4 DAE. The 5-day-old accessory glands were fully mature (Fig. 2F-J).

### Spermatogenesis

**Fig. 3:**
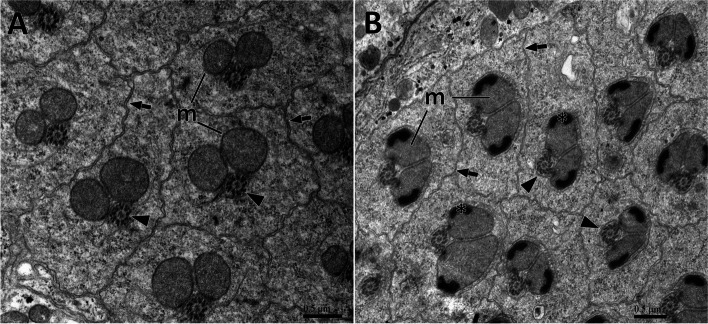
Cross-section through early spermatids of *C. pallens*. **A** Cross-section through early spermatids. Note two round mitochondrial derivatives (m) and the axoneme consisting of a bundle of microtubule (arrowhead). Arrows indicate plasma membrane. **B** Cross-section through early spermatids with partially condensed mitochondrial derivatives. Each shows microtubule triplets (arrowhead) and two mitochondrial derivatives (m) with a partial crystallization of the inner material (asterisk). Arrow indicates plasma membrane

Early seminal cysts are filled with spermatids separated by a cytomembrane. Each cell envelope contained an axoneme consisting of a bundle of microtubules and two circular mitochondrial derivatives, which were about 0.5 μm in diameter and had good symmetry (Fig. 3A). The two mitochondrial derivatives are irregularly shaped and pressed close to each other to form narrow boundaries. The two far ends progressively condensed and initiated crystallization (Fig. 3B).

**Fig. 4:**
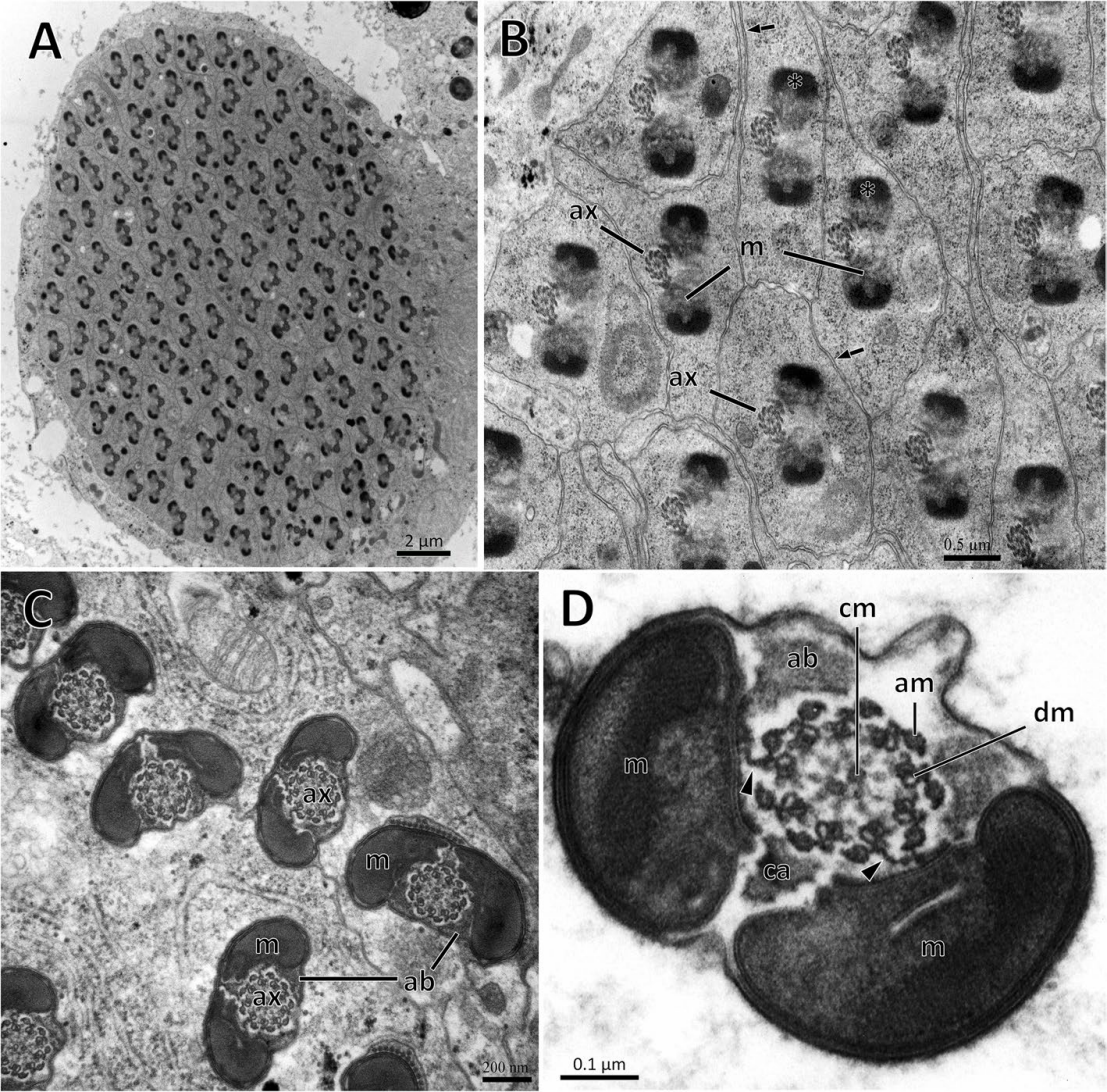
Cross-section through tails of *C. pallens* spermatids. **A** Cross-section through a cyst showing about 128 flagellums of maturing spermatids. **B** Magnification of spermatids flagellums before maturation. **C** Cross-section through flagellums of matured spermatids. **D** Details of a matured spermatid. The axoneme showed a 9 + 9 + 2 pattern. m, mitochondrial derivative; ab, accessory body; ax, axoneme; dm, doublet microtubule; am, accessory microtubules; cm, central microtubule; ca., centriole adjunct. Asterisk indicates initial paracrystalline material. Arrows indicate plasma membrane. Note the bridges (arrowheads) anchoring the axonemal 2 and 5 to the mitochondrial derivatives

After meiosis, a seminal cyst containing approximately 128 spermatids tightly surrounded by a plasma membrane was observed (Fig. 4A). Crystallization at both ends of the expanded mitochondrial derivative. A gap appeared between the two mitochondrial derivatives, leaving room for the future insertion of the axoneme. The microtubules were arranged more regularly and the axoneme, located on one side of the two mitochondrial derivatives (approximately 1.1 μm in length), was well defined. Cross-sectioned sperm flagella exhibited pronounced bilateral symmetry (Fig. 4B). In mature spermatids, the cytoplasm was eliminated. The axoneme is surrounded by a layer of centriole adjunct material and forms two elongated accessory bodies. The axoneme was located along the middle region of the flagellum, in the space between two symmetrical accessory body/mitochondrial derivative sets (Fig. 4C). In the flagellum of mature spermatids, the axoneme exhibited a typical 9 + 9 + 2 pattern, including nine doublet microtubules (dm), nine accessory microtubules (am) and two central microtubules (cm). Two bridges linked the second and fifth axonemal doublets to the inner wall of mitochondrial derivatives (Fig. 4D).

**Fig. 5:**
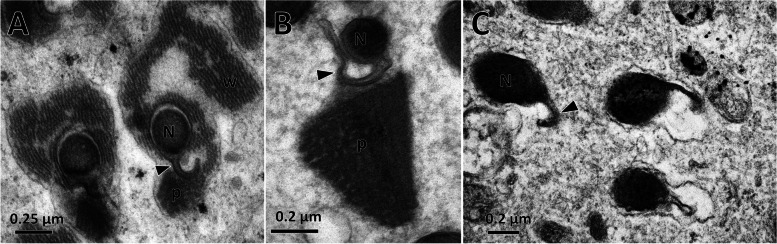
Cross-section through spermatid nuclei of *C. pallens*. **A** Cross-section of spermatids surrounded by parallel waves (w) and paracrystalline materials (p). **B** Cross-section of a spermatid accompanied by triangular paracrystalline materials. **C** Cross-section of spermatid after individualization. N, nuclei. Arrowhead indicates tail-shaped wing

In the heads of early spermatids, the nucleus had a cylindrical shape (0.4–0.5 μm in diameter) and tapered towards the anterior end. During this period, spermatid bundles were immersed in the cytoplasm of the cyst cells. The anterior spematid region is encircled by parallel crystalline waves and paracrystalline materials. In cross-section, the nucleus envelope extended towards the paracrystalline region in a tail-shaped wing (approximately 0.25 μm long), appearing tadpole-like morphology (Fig. 5A). Posteriorly, the parallel crystalline waves disappeared, but the paracrystalline materials remained adjacent to the nuclear wings (Fig. 5B). Furthermore, these two structures disappeared, whereas the tail-shaped wings were maintained (Fig. 5C).

**Fig. 6:**
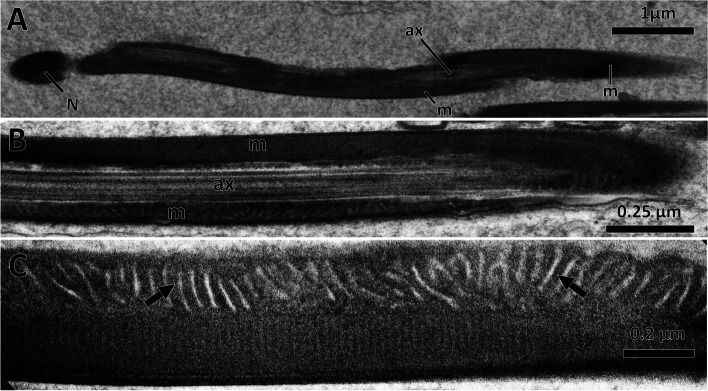
Longitudinal section of the spermatids of *C. pallens*. **A** Longitudinal sections of a spermatid showing an elliptical nucleus and a long flagellum. **B** Magnification of a spermatid flagellum. **C** Mitochondrial derivatives with the arranged cristae (arrow). N, nucleus; m, mitochondrial derivative; ax, axoneme

The elongated spermatids were approximately 8 μm long, consisted of an elliptical head, a long flagellum, and a transition region between them (Fig. 6A). The axoneme and two mitochondrial derivatives in the flagellum were easily distinguishable in longitudinal sections (Fig. 6B). At regular intervals, many peripheral cristae were observed perpendicular to the axis of the mitochondrial derivatives (Fig. 6C).

### Ultrastructure of testis and microsporidian spores

**Fig. 7:**
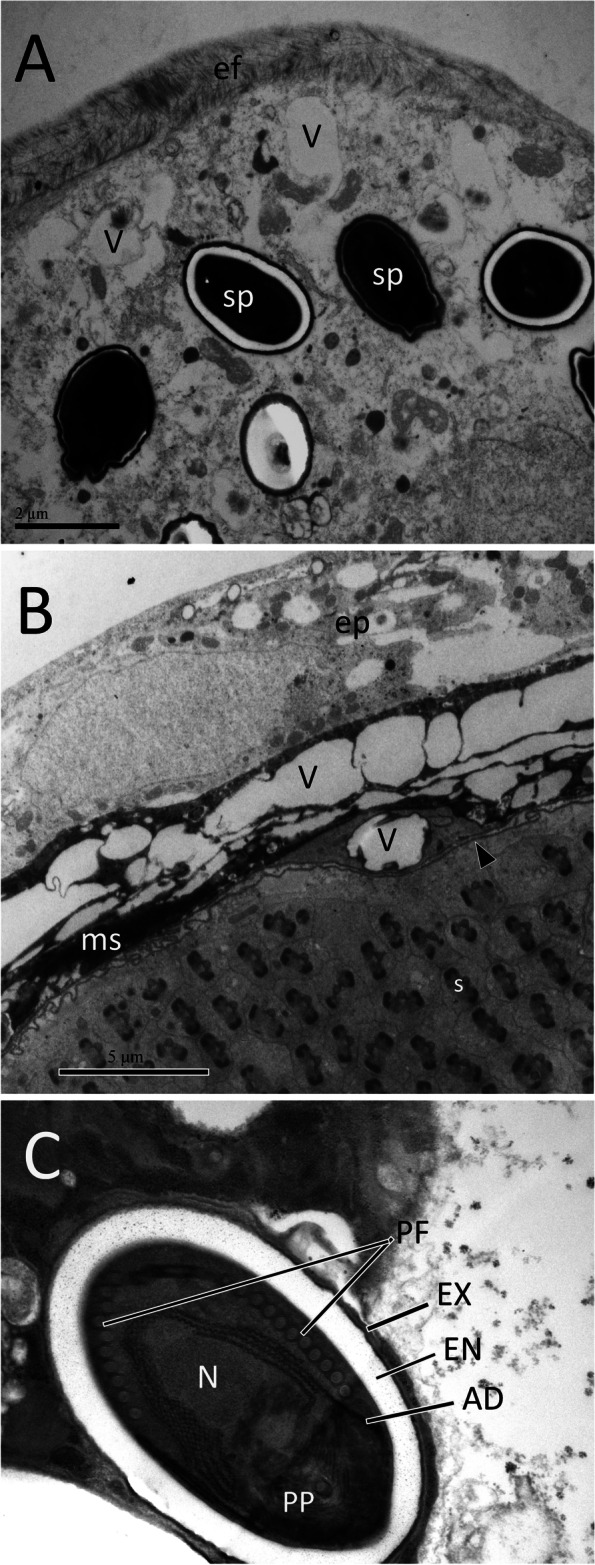
Ultra structure of *C. pallens* testis. **A** A seminal cyst covered with a layer of extracellular fibers (ef). **B** A cyst containing spermatids (s). **C** Mature spore with a well-developed wall consisting of an exospore (EX) and endospore (EN). V, vesicle. SP, spore. ep, epithelium. ms, muscular-connective sheath. AD, anchoring disk. PF, polar filament. PP, lamellar polaroplast. N, nucleus. Arrowhead indicates basal lamina

In the cross-section of *C. pallens* testis, the seminal cysts were covered with a layer of extracellular fibers. There were many microsporidian spores in the cytoplasm of the epithelial cells (Fig. 7A). The wall of the seminal cysts consists of a layer of epithelium, a muscular-connective sheath, a basal lamina, and vesicles of different sizes. The lumens of the seminal cysts were filled with spermatozoa (Fig. 7B). Microsporidium is widely distributed throughout the testicular epithelium. The mature spores were rounded or oval (1.4–3 μm in diameter or length), with a lamellar polaroplast and an anchoring disk with relatively long arms. The isofilar polar tube was arranged into seven or eight coils in one row (Fig. 7C).

**Fig. 8:**
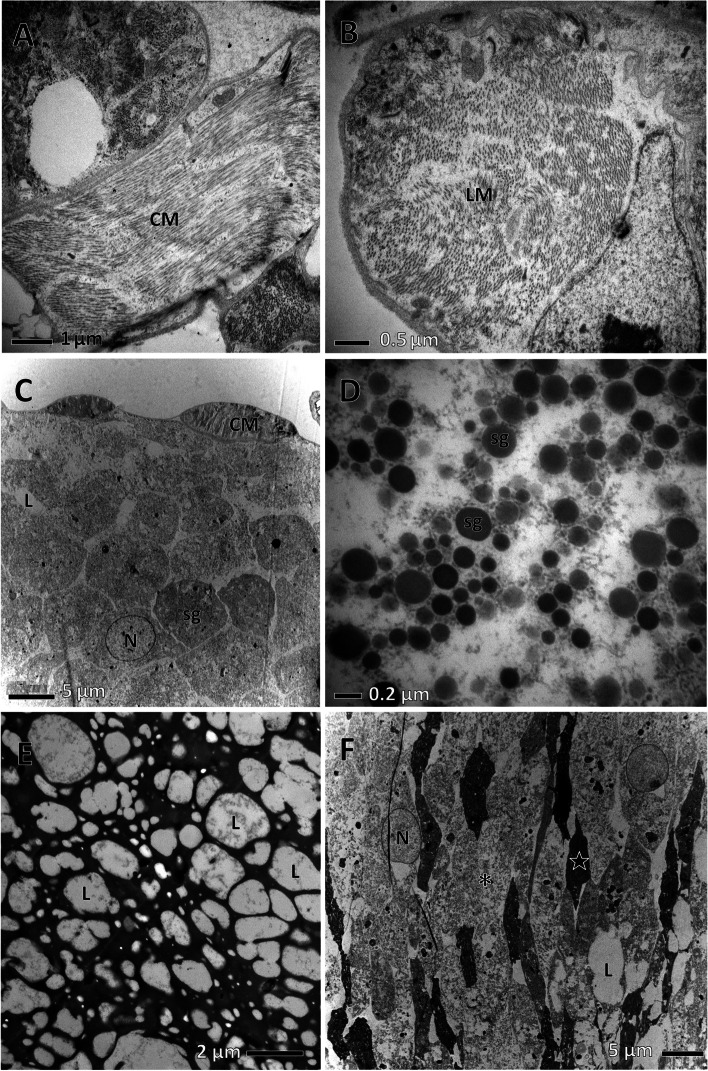
Ultrastructure of *C. pallens* accessory glands. **A** Cross-section of accessory glands, showing circular muscle fibers (CM). **B** Details of longitudinal muscle fibers (LM). **C** Closely arranged secretory granules (Sg) of big size. **D** Scattered secretory granules (Sg). **E** Secretory epithelium (ep) with a network structure. **F** Densely packed secretory materials characterized by compaction of the moderated (asterisk) and heavy electron-dense materials (star). V, vesicle; L, lumen; N, nucleus

Across sections of the accessory glands showed many longitudinal and circular muscle fibers (Fig. 8A, B). A large number of secretory granules are visible in the secretory epithelium. They were scattered or closely arranged, round or irregularly shaped, and varied in size from 0.2 to 8 μm (Fig. 8C, D). Numerous tiny vesicles were dispersed in the cytoplasm of the epithelial cells, forming a network (Fig. 8E). In some sections, moderately electron-dense secretory materials were closely aligned with strips of heavy electron-dense secretory materials (Fig. 8F).

## Discussion

The features of the insect reproductive system may offer additional taxonomic characteristics and assist in elucidating the phylogenetic relationships between various groups. In addition, they may reflect physiological, ecological, and behavioral differences, none of which have been thoroughly clarified in Chrysopidae. To date, the morphology of the male reproductive system has been described in only a few Chrysopidae species. However, its fine structure remains unclear, as most studies were performed before the 1970s, when reliable technical approaches such as transmission electron microscopy (TEM) were not available [22]. In the present study, we comprehensively described the anatomy and ultrastructure of the *C. pallens* male reproductive system and its spermatogenesis using light microscopy and TEM. We aim to lay the foundation for future systematic, phylogenetic, and reproductive physiological studies.

Previous studies have demonstrated that the general structure of insect reproductive systems is consistent across the same family. However, morphological details may vary among species, particularly in attributes such as the size, number, shape, and structure of reproductive organs and organelles [23, 24]. This phenomenon was also observed in this study. The male reproductive system of *C. pallens* is morphologically similar to that of *Chrysopa oculata*, another species of Chrysopidae, with a pair of testes, slim vas deferens, seminal vesicles, accessory glands, and a short ejaculatory duct [22]. Furthermore, the sperm ultrastructure of *C. pallens* resembles that of *C*. *formosa* and *Dichochrysa prasina* [16]. However, obvious differences in the macrostructure of the accessory glands and the ultrastructure of spermatids also exist between *C. pallens* and other Chrysopidae species. For example, in the accessory glands of *C. pallens*, the anterior glandular lobe was triangular, and the seminal vesicle is irregular. Nevertheless, these two components in *C. oculata* are virgulate and elliptical, respectively [22].

The sperm ultrastructure of *C. pallens* resembles that of *D. prasina* and *C. formosa*, with the following similarities:1. a single axoneme; 2. pairs of bilateral accessory bodies, 3. a pair of bilateral mitochondrial derivatives, 4. a circular nucleus accompanied by a triangular paracrystalline layer, and a 5. nucleus envelope expansion in thin wings. However, the sperm of *C. pallens* also have unique features. First, in mature spermatids of *C. pallens*, both mitochondrial derivatives and their extensions towards each other are condensed. However, in mature spermatids of *D. prasina* and *C. formosa*, the two mitochondrial derivatives are condensed and their extensional areas are not[16]. Second, in *C. pallens*, the spermatid nucleus was surrounded by linear material that appeared as parallel waves (Fig. 5A), whereas in *D. prasina* and *C. formosa*, the corresponding material consisted of discontinuous granules separated from each other [16]. Finally, in *C. pallens* spermatids, both the parallel waves and paracrystalline materials progressively diminish and are eventually eliminated from the sperm. Nevertheless, at sperm maturity in *D. prasina* and *C. formosa*, the granule material disappears and the crystallized material still adheres to the nuclear wings [16]. Structures surrounding the sperm nucleus in neuropterans have also been described in other species. For example, during the elongation of spermatids in *Kolla paulula*, the nucleus is encircled by microtubules that shape the nucleus in hemipteran species [13]. In the species studied here, the parallel waves surrounding the nucleus may play similar roles.

In *Drosophila melanogaster*, spermatogonia produced by germline stem cells undergo four mitotic and two meiotic divisions to generate cysts containing 64 spermatids [25, 26]. In the present study, we found that *C. pallens* spermatogonia underwent one or more mitotic divisions to produce cysts with 128 spermatids, which corresponded to 2^7^ spermatogonial divisions.

Prior to this study, there have been no reports on the ultrastructure of the male reproductive organs of Neuroptera species. Therefore, we could only compare the testis and accessory gland ultrastructures of *C. pallens* with those of non-Neuroptera species. A typical feature of *C. pallens* testes is the extensive distribution of microsporidian spores in the testicular epithelium (Fig. 7A). Lacewings are known for their symbiotic association with yeasts, bacteria and microsporidium [18]. Microsporidian spores have also been observed in the digestive organs of *C. carnea*. In this study, they were identified in the testicular epithelium of *C. pallens*. In most insects, the male accessory gland assists in the shift of sperms from male to female by secreting seminal fluid and producing spermatophores [19]. In keeping with their functions, insect accessory glands have evolved to possess common structural features. In the present study, we found that the accessory gland ultrastructure, consisting of muscular fibers, basal lamina, epithelium, and lumen in *C. pallens*, was similar to that in leafhoppers (Hemiptera) [13]. In addition, the epithelial cells in both species contained several types of secretory granules of diverse sizes that were stained. A similar constitution led us to conclude that secretion in *C. pallens* accessory gland belongs to a common apocrine mechanism in insects, which is pooled from epithelial cells to the tubular lumen backed by the apical membrane [27–29]. However, insect accessory glands may vary strikingly among taxa based on their detailed structures [2, 5–11, 13, 14, 30, 31]. In the present study, the *C. pallens* accessory gland showed distinctive traits, particularly the presence of many circular and longitudinal muscle fibers (Fig. 8A, B). We speculate that this is structural compensation for the lack of a long common ejaculatory duct. This reflects the reliability of *C. pallens* accessory glands in spermiation.

## Data Availability

Not applicable.
